# Translation, Cultural Adaptation, and Content Validation of the Palliative Care Self-Efficacy Scale for Use in the Swedish Context

**DOI:** 10.3390/ijerph19031143

**Published:** 2022-01-20

**Authors:** Sofia Andersson, Lisa Granat, Margareta Brännström, Anna Sandgren

**Affiliations:** 1 Center for Collaborative Palliative Care, Department of Health and Caring Sciences, Faculty of Health and Life Sciences, Linnaeus University, 351 95 Växjö, Sweden; lisa.granat@lnu.se (L.G.); anna.sandgren@lnu.se (A.S.); 2 Department of Nursing, Campus Skellefteå, Umeå University, 931 87 Skellefteå, Sweden; margareta.brannstrom@umu.se; 3 The Arctic Research Centre, Umeå University, 901 87 Umeå, Sweden

**Keywords:** content validity, end-of-life, face validity, healthcare professionals, palliative care, PCSE scale

## Abstract

The Palliative Care Self-Efficacy Scale (PCSE) is a valid instrument in English for assessing healthcare professionals’ self-efficacy in providing palliative care; it has not been translated into Swedish. The aim of this study was to describe the translation, cultural adaptation, and content-validation process of the PCSE scale. In this study, forward and backward translations, pretesting including an expert panel (*n* = 7), and cognitive interviewing with possible healthcare professionals (physicians, nurses, and assistant nurses) (*n* = 10) were performed. Experts in palliative care rated items on a Likert scale based on their understandability, clarity, sensitivity, and relevance. The item-level content validity index (I-CVI) and modified kappa statistics were calculated. Healthcare professionals were interviewed using the think-aloud method. The translation and validation process resulted in the final version of the PCSE scale. The average I-CVI for sensitivity was evaluated and approved. Most of the items were approved for clarity, some items lacked understandability, but a majority of the items were considered relevant. The healthcare professionals agreed that the items in the questionnaire evoked emotions, but were relevant for healthcare professionals. Thus, the Palliative Care Self-Efficacy scale is relevant for assessing healthcare professionals’ self-efficacy in palliative care in a Swedish care context. Further research using psychometric tests is required.

## 1. Introduction

Palliative care can be complicated and challenging for healthcare professionals (HCPs) [1]. The coronavirus (COVID-19) pandemic in 2019 highlighted the need for increasing the knowledge of palliative care in residential care homes [2,3]. All HCPs should be educated and trained in palliative care to ensure appropriate symptom management [4]. A study conducted in different settings (nursing homes, mental health facilities, and care homes) showed that factors associated with high self-efficacy in HCPs were the setting, age (older), gender (female), formal palliative care training, and knowledge of palliative care [5]. 

Knowledge of palliative care and self-efficacy can be relevant measures for education and palliative care intervention as they can influence caretaking and personal behavioral change [1]. If HCPs believe that they can provide palliative care and improve a patient’s quality of life, they will be more likely to deliver palliative care to the patient [6]. 

According to Bandura’s social cognitive theory regarding self-efficacy, the outcomes that are integral to human motivation, including perseverance, resiliency, and adaptation, are central to the confidence individuals have in their ability to perform a specific behavior or skill. Based on this theory, self-efficacy refers to our overall belief in organizing, executing, and succeeding. If HCPs have positive emotions, they can generate greater feelings of self-efficacy. Self-efficacy can affect how much effort HCPs will spend on a task, how long they will persevere when experiencing difficulties, and how resilient they will be in difficult situations. Higher self-efficacy can also improve decision-making and information sharing [6]. Self-efficacy is important for behavioral changes in HCPs, and for the evaluation of training and education [7]. 

One instrument to measure HCP’s self-efficacy in palliative care is the Palliative Care Self-Efficacy (PCSE) Scale [1], which is a further development of the instrument “Palliative care providers’ views and attitudes” [8]. The PCSE scale has two theoretical dimensions: psychosocial support and symptom management. It evaluates an individual’s beliefs in themselves, including self-confidence, self-reliance, and assurance, related to providing palliative care [1]. The PCSE scale has been translated and used in hospitals in Iran to evaluate outcomes after education in palliative care, and self-efficacy has been tested before and after an intervention in educational workshops. The results showed that nurses’ perceived self-efficacy scores improved [9]. Another study also used the PCSE-scale and found increased self-efficacy among nurses after a palliative care education intervention [10]. However, to the best of our knowledge, there are no instruments in the Swedish context to assess healthcare professionals’ self-efficacy in palliative care. 

### Aim

The aim of this study was to describe the translation, cultural adaptation, and content validation process of the Palliative Care Self-Efficacy scale to assess healthcare professionals’ self-efficacy in providing palliative care in the Swedish context.

## 2. Method 

### 2.1. Preparation 

Permission to translate, culturally adjust, and validate the PCSE scale in Swedish was obtained from the original developer [1]. 

#### The Instrument: PCSE Scale

The PCSE scale comprises 12 items in which participants are asked to rate their self-efficacy in palliative care [1]. The scale includes two theoretical domains. Items 1–6 comprise questions about psychosocial support and items 7–12 comprise questions about symptom management. Participants rate their self-efficacy based on their perceived degree of confidence with patient and family interactions, and patient management topics, by ticking the relevant box on a four-grade scale; 1—needs further basic instructions, 2— confident to perform with close supervision/coaching, 3—confident to perform with minimal consultation, or 4—confident to perform independently. The PCSE scale has been tested in residential care homes with nurses and nursing assistants for validity and reliability with strong consistency [1].

## 3. Translation Process

In this study, the World Health Organization’s (WHO’s) process of translation and adaptation of instruments was followed. The process included forward and backward translations, pre-testing including expert panels (experts in palliative care), and cognitive interviewing (think-aloud interviews) with possible participants (HCPs) [11]. 

### 3.1. Step 1: Forward Translation

Initially, the PCSE scale was forward translated into Swedish, to retain the meaning of the English text as much as possible, by an English linguistics researcher and a qualified English school teacher; they were not aware of the questionnaire before. All four authors also prepared a forward translation of the PCSE scale into Swedish in the Swedish context of palliative care. A direct translation was not possible because some words needed to be changed to match the Swedish language and context. The authors had meetings to identify and discuss potentially unclear text, and agreed on the first version of the translated version of the PCSE scale. 

### 3.2. Step 2: Backward Translation

The PCSE was then back translated into English by two independent persons. One researcher and one professional editor, both of whose mother tongue was English; they had a good knowledge of the Swedish language. They had no prior knowledge of the questionnaire. The authors had a meeting to identify and correct the unclear text. They also compared the original and the two backward-translated versions, along with the first Swedish translation. 

### 3.3. Step 3: Pre-Testing

This step included both pre-testing with researchers in palliative care (3.3.1) and cognitive interviews with HCPs (3.3.2).

#### 3.3.1. Pre-Testing 

The Swedish version of the PCSE scale was sent via e-mail to seven researchers in palliative care (hereafter referred to as experts). They belonged to different professional backgrounds: two nurses/professors, two nurses/associate professors, two physicians/professors, and one social worker/associate professor. The experts represented different genders (there were four women and three men) and were aged between 59–65 years. They were recruited from different networks during October 2020. The experts were asked to rate the questionnaire’s items using a Likert-scale (1–5), regarding the clarity, understanding, relevance, and sensitivity of the scale. The experts were also asked to rate and respond to the questionnaire as a whole. They were asked to consider the alternative wordings for every item’s responses. For clarity, answers in categories 1–3 were combined to form a new category 0 (not clear), and categories 4–5 were combined to form a new category 1 (clear). For the understandability category, 1–3 were combined to form a new category 0 (not understandable), and category 4–5 were combined to form a new category 1 (understandable). For sensitivity, categories 1–3 were combined to category 0 (very sensitive), and 4–5 were combined to category 1 (not sensitive). For relevance, categories 1–3 were combined to 0 (not relevant), and categories 4–5 were combined to category 1 (relevant). 

The item-level content validity index (I-CVI) was used to examine the content validity of the scale [12]. The number of experts giving a rating of 1 (clarity, understandability, sensitivity, and relevance) was divided by the total number of experts, which resulted in the proportion of experts in agreement. If there were fewer than five experts, the I-CVI 1.0 was considered as valid. If there were six experts or more, an I-CVI over 0.78 was considered valid. The average I-CVI focused on the average item by summing the I-CVI and dividing it by the number of items [12].

To validate the I-CVI on relevance, the modified kappa was calculated to provide information about the degree of agreement beyond chance (with kappa designating agreement on relevance (I-CVI-pc)/(1-pc). Kappa values were as follows: fair = 0.40–0.59, good = 0.60–0.74, and excellent >0.74 [12,13]. The experts’ free answers were valuable in the translation process and constituted an essential part of the qualitative review of an item. The free answers also provided suggestions for other formulations of the items. The authors had another meeting to identify any potential lack of clarity and they agreed on the third version of the PCSE scale.

#### 3.3.2. Cognitive Interviewing 

Cognitive interviewing was conducted using the think-aloud method [14] with HCPs. Cognitive interviewing provided information about translation and how the questions were being understood. Cognitive testing of translated questions could uncover difficulties faced by participants when interpreting a question [15]. 

The participants (*n* = 10) were recruited from different care settings (nursing homes, home care, medicine wards, oncology wards, intensive care, and pre-hospital care). All participants were experienced in caring for persons with palliative care needs. The interviews were separately conducted by two authors (S.A. and L.G.) during December 2020 through four face-to-face interviews and six digital interviews (due to the COVID-19 pandemic and social distancing regulations). At the time of the interviews, the participants received information about the study. The participants provided informed consent before the interviews were recorded. 

First, the participants were asked about their age, gender, employment, education, and palliative care experience. Face validity was measured through face-to-face interviews with the participants (Table 1). Each item of the PCSE scale was displayed to the participants so they could respond spontaneously and describe what they thought about the item. The participants were also asked to respond to the questionnaire as a whole and to review the items based on their clarity, understanding, sensitivity, and relevance. Participants were also asked if they had suggestions for improving the translation or alternative word suggestions. Furthermore, the participants were asked if they thought the questions were a good fit in Swedish care settings. Suggestions for a better fit with Swedish culture were elicited. All interviews were audiotaped and summarized according to each item [11]. The authors further identified any potential lack of clarity and agreed on a fourth version of the PCSE scale. 

### 3.4. Step 4: The Final Version

After the pre-testing period, the authors identified some potential lack of clarity and agreed on a fourth and final version of the PCSE scale. The authors discussed the original PCSE scale, results from pre-testing, and cognitive interviews until a consensus was reached. Some items were reworded to make them more understandable, but care was taken to keep items as close to the original version as possible. 

### 3.5. Cultural Adaption

Cultural adaptions were made during every step (1–4) of the translation process by removing background characteristic information about ethnicity and religion, as they are considered to be sensitive information under Swedish law. During the translation process, the wording of some items was also changed to better fit the Swedish context, but they were not changed significantly so that the translated version still matched the original PCSE scale. 

## 4. Results

The translation and validation processes are presented in four steps: forward translation, backward translation, pre-testing with experts and cognitive interviews, and the final version. 

### 4.1. Step 1: Forward Translation

In step 1, the English version was translated into Swedish. For cultural adaptation, questions about the background characteristics of the participants regarding ethnicity and cultural background were removed in the forward translation. These questions were not appropriate for use in the Swedish care context. In response, in alternatives two, three, and four, the word “confident” was changed to “ability”. After the forward translation, the authors discussed the difficulties they faced while translating the term “queries” in item 6 to Swedish. The authors decided to use a Swedish word that correlated to the English word “questions”. The word “coping” was also discussed, but the authors decided to retain it because it best fitted the Swedish context. 

### 4.2. Step 2: Backward Translation

In Step 2, the Swedish version was translated back into English. In item 3, the word “people” was changed to “patients/relatives”. The translation of item 4, “Discussing different environmental options (e.g., hospital, home, and family)” was changed to “Discussing different care options (e.g., hospital, nursing home, and home)” to better fit the Swedish context (Table 2). In item 7, the word “reacting” was changed to “responding”. In items 8–12 the word “reacting” was changed to “pay attention to”. In item 8, the word “terminal” was changed to “end-of-life”. 

### 4.3. Step 3: Pre-Testing with Experts and Cognitive Interviewing 

#### 4.3.1. Pre-Testing with Experts

The seven experts gave their comments on the questionnaire and rated each item’s understandability, clarity, sensitivity, and relevance. The average I-CVI was 0.64 for understandability and 0.74 for clarity, which was interpreted as not approved. The average I-CVI for sensitivity was 0.83, which was interpreted as approved. Based on the calculations, the items of the scale lacked understandability, and only two out of the twelve items were approved. Most of the items were approved for clarity based on the I-CVI. Most of the items were calculated to be insensitive (Table 3). 

The average I-CVI for relevance for all items was calculated to be over 0.94, and the kappa value for change of agreement was calculated to be more than 0.81, which was evaluated as excellent (Table 4).

The content validation of the experts’ ratings and answers revealed that the PCSE scale comprised important and essential questions. The items were assessed as relevant in the Swedish care context and included several important aspects of care for patients with palliative care needs.

*“It is a nice scale, and the questions are relevant. Overall, the questionnaire is easy to understand, clear, and relevant, and will not provoke emotions”*.

(expert 6)

However, some of the items needed some revisions, and the items were rewritten according to the experts’ suggestions. In the first domain, three items were changed (items 1, 5, and 6), and in the second domain, two items were changed (items 7 and 12). Item 1 was described as a general question that could be difficult for HCPs to answer. In item 1, the word the “dying process” was changed to “the dying”.

*“Now, questions are directed to care professionals and then we can see that it is a process, but how do we see it as a process (item 1)? Would it be enough with the word dying?”*.

(expert 3)

Regarding item 5, the experts said that it was a bit difficult to understand what and with whom they should discuss regarding patients’ wishes after their death (Table 4).

*“Something is unclear with this formulation. You do not have so many wishes when you are dead, but you can have thoughts about wishes of what is going to happen after your death”*.

(expert 1)

#### 4.3.2. Cognitive Interviewing 

Overall, the ten participants mentioned that the PCSE scale was clear, understandable, and relevant for evaluating HCP self-efficacy in palliative care in the Swedish care context. However, the participants had some suggestions to make some items clearer and more understandable; in total, four items were rewritten in both subscales (psychosocial support and symptom management). The participants thought that the items could be sensitive, but these feelings were described as understandable in palliative care situations. However, these items were important to reflect upon in the care of patients with palliative needs.

*“Interesting, and self-explorative… Yes, it could evoke thoughts that this could be a subject that I need to get deeper into. You see gaps in your knowledge when you are asked these essential questions*”.

(HCP6)

In item 3, it was unclear what the word “support service” referred to, so the word “service” was excluded. The participants thought that item 4 needed more clarity and examples of different care settings in the item, and whom the HCPs should discuss with. Several participants thought that item 5 needed more clarity regarding with whom and how the HCPs should discuss the patient’s wishes after their death (Table 4).

*“What are you thinking about ‘What is going to happen after their death?’ Are you thinking of ‘How is my funeral going to be?’”*.

(HCP2, regarding item 5)

### 4.4. Final Version

In summary, the experts and the HCPs agreed that the items in the questionnaire could be sensitive and evoke emotions, but they were important and relevant for HCPs. Overall, both the experts and HCPs described that the items in the PCSE scale were understandable and clear. The alternatives for information text about the scale, about confidence level in 12 patient/family interactions, and clinical management issues were changed in the final version. The word “preform” was removed from the responses in options two, three, and four. In response in option two, the word “close” was removed. Items 2, 3, 4, 5, 7, 8, 9, 10, and 11 were rewritten based on the answers and comments in step 3 (Table 4). In item 2, the word “upset” was changed to “emotional”. In item 3, the word “service” was removed. In items 4 and 5, the word "discussing” was replaced by “conversation”. In item 7, the word “responding” was changed to “pay attention to.” In item 8, the word “coping” was replaced by “remedy”. In items 9, 10, and 11, the word “coping” was replaced by “assess and remedy”.

## 5. Discussion

In this study, we developed the first Swedish version of the PCSE scale by following the WHO’s translation process with experts and HCPs [11]. The participants in this study mentioned that the PCSE scale could be helpful for HCPs to reflect on their care services for patients with palliative care needs in the Swedish care context. During the translation process, close attention was paid to ensuring that the translated version matched the original version of the PCSE scale to ensure validity. In this study, the translation of the Swedish version of the PCSE scale was tested for content and face validity. In an earlier study, an English version of the PCSE scale was tested for construct validity and internal consistency, and the results showed that the scale was valid and reliable with strong consistency [1]. Another study performed in hospitals in Iran aimed to study the effect of palliative care training on perceived self-efficacy of the nurses [9]. The scale was also tested for content and face validity using the judgment of experts, qualitative validity, and Cronbach’s alpha. For the first six items, Cronbach’s alpha was 0.848, and for the next six items, Cronbach’s alpha was 0.780; the overall Cronbach alpha was 0.704 [9]. In a study by Philips et al. [1], HCPs were employed in a community in Australia. Their study aimed to investigate the psychometric properties of the `Palliative Care Self-efficacy scale’, an instrument designed to assess nurses, enrolled nurses, and care assistants’ degree of confidence in engaging in patient and family interactions at the end-of-life. The participants in this study had different professions, such as physician, nurses, nurse assistant, and were employed in communities and regions in Sweden. The results showed that some of the items were not approved based on understandability, but 11 of the 12 items were considered as excellent based on relevance. 

Some background characteristics that were present in the original PCSE scale, were not appropriate for use in the Swedish context. For example, questions about ethnicity and culture are related to background characteristics. During the translation process, some words that were inappropriate in the Swedish care context were also changed. However, as the PCSE scale was developed in English, there was some lack of clarity related to the translation of questions. These arose from translation choices, cultural factors influencing interpretation (even in a perfectly translated question), and a lack of overlap between the language/culture of the translation and language [15].

The original PCSE scale evaluates self-efficacy in palliative care for nurses and assistant nurses [1]. In this study, we explored the content validity of the PCSE scale for nurses and nurse assistants, as well as for physicians. In palliative care teamwork is important [16,17], so in this study we decided to also include physicians. However, it is important to highlight that this scale evaluated HCP’s self-efficacy and not competence [18]. Earlier studies that measured the effects of a palliative course through self-efficacy have shown a strong ceiling effect, which means that caregivers rated their self-efficacy as continuously high [7] and assessed more than self-efficacy through communication, patient management, and multidisciplinary teamwork [19]. Another study reported that higher self-efficacy was associated with spiritual care competence [20]. Self-efficacy was also found to be lower if nurses were older and had more work experience. This was explained by the complex need for palliative care [21]. During the translation process, physicians, nurses, and assistant nurses were included as experts and HCPs. Therefore, the results indicated that different HCPs could use the PCSE scale in both community and regional care contexts.

Earlier studies have shown that palliative care training could improve nurses’ self-efficacy [9,10] and the symptom management of perceived self-efficacy [9]. HCPs trained in palliative care were more likely to have higher self-efficacy in discussing end-of-life issues with residents and families at nursing homes [5]. Previous studies have also reported that HCP’s confidence can be improved if they receive palliative care training [22,23]. HCPs must believe that their actions will lead to a positive outcome and believe they have that capability [1]. With the PCSE scale, it is possible to evaluate whether HCPs need education in different care contexts for patients with palliative care needs. The PCSE scale has been used in other studies for pre- and post-palliative care planning [9,10] and for comparing intervention groups for education in palliative care and control groups [10]. 

Translating, adapting, and validating an instrument into another language and context is a complicated process, which may take several years, and usually includes several studies [24]. Several guidelines exist that describe different ways to translate and culturally adapt instruments e.g., [11,25]. Earlier studies have also made partial validation of instruments in order to translate and culturally adapt instruments [26,27]. However, there is a need for further studies to evaluate the PCSE scale with regards to other psychometric tests with HCPs in different settings.

### Methodological Considerations

The design, method, and analysis of the current study can be assumed to be relevant, as our aim was to describe the translation, cultural adaptation, and content validation process of the PCSE. One strength was that we included both experts in palliative care and HCPs, with different experiences of palliative care. In the analysis, we used both quantitative and qualitative methods to obtain a broader understanding of the participants’ reflections. To ensure credibility in the translation, we attempted to remain as close to the original English text as possible. However, a direct translation was not possible because some words needed to be changed to match the Swedish language and context. The translation process was verified with original quotations from both experts’ comments and interviews with HCPs. Seven experts were invited to participate; however, one of the experts had only included written comments and no ratings on the understandability, clarity, sensitivity, or relevance of the items. This was because the expert reported that it was difficult to rate the items on a scale; therefore, they provided a description instead. A strength of this study was the detailed description of the steps undertaken, according to WHO guidelines (WHO), for the PCSE scale in Swedish. 

## 6. Conclusions

The results of this study indicated that the PCSE scale is relevant and usable for assessing HCP’s self-efficacy in palliative care in a Swedish care context. Furthermore, even though the scale seemed relevant, more work has to be performed to improve the understandability and clarity of the items.

## Figures and Tables

**Table 1:** Background characteristics.

Characteristics	Total
Gender n (%)	
Male	4 (40)
Female	6 (60)
Age (years)	
Median	56.5
Mean	54.8
Range (min–max)	33–75
Profession n (%)	
Assistant nurse	3 (30)
Registered nurse	4 (40)
Physicians	3 (30)
Previous experience in palliative care *n* (%)	
Yes	10 (100)
Time spent in profession (years)	
Median	28
Mean	28.5
Range (min–max)	12–51

**Table 2:** English original, step 1–2 forward/backward translation, step 3 pre-testing, and step 4 final version.

Items PCSE Scale	English Original	Step 1–2 Forward/Backward Translation	Step 3 Pre-Testing	Step 4 Final Version
4	Discussing different environmental options (e.g., hospital, home, family)	Diskutera olika vårdalternativ (sjukhus, vård- och omsorgsboende, eget hem)	No change	Samtala med patienter om olika vårdalternativ (sjukhus, vård- och omsorgsboende, eget hem)
5	Discussing patient’s wishes for after their death	Diskutera med patienten om önskemål efter dennes död	Diskutera med patienten om önskemål om vad som ska ske efter dennes död	Samtala med patienter om önskemål om vad som ska ske efter dennes död
6	Answering queries about the effects of certain medications	Besvara frågor om effekter av vissa läkemedel	Besvara frågor om effekter av läkemedel	No change

**Table 3:** The understandability, clarity, and sensitivity of items on the PCSE scale; dichotomized into 1–3 = 0 and 4–5 = 1.

Items PCSE Scale	Understandability			Clarity			Sensitivity		
	Total Experts	I-CVI	Evaluation	Total Experts	I-CVI	Evaluation	Total Expert	I-CVI	Evaluation
Item 1	4/5	0.80	Approved	4/5	0.80	Approved	3/5	0.60	
Item 2	4/6	0.67		4/6	0.67		5/6	0.83	Approved
Item 3	4/6	0.67		5/6	0.83	Approved	5/6	0.83	Approved
Item 4	5/6	0.83	Approved	5/6	0.83	Approved	5/6	0.83	Approved
Item 5	4/6	0.67		4/6	0.67		2/6	0.33	
Item 6	4/6	0.67		5/6	0.83	Approved	6/6	1	Approved
Item 7	2/6	0.33		3/6	0.50		5/6	0.83	Approved
Item 8	4/6	0.67		5/6	0.83	Approved	6/6	1	Approved
Item 9	4/6	0.67		5/6	0.83	Approved	6/6	1	Approved
Item 10	4/6	0.67		4/6	0.67		5/6	0.83	Approved
Item 11	4/6	0.67		5/6	0.83	Approved	5/6	0.83	Approved
Item 12	2/5	0.40		3/5	0.60		5/5	1.00	Approved
Average I-CVI	0,64			0.74			0.83		

Item-level content validity index (I-CVI), >0.78 approved; Average item-level content validity index = average I-CVI.

**Table 4:** Relevance of the PCSE scale: items, expert ratings, and agreement.

Item PCSE Scale (12) Relevance	Number of Experts	Number Giving a Rating of 4–5	I-CVI	Pc ^a^	Kappa ^b^	Evaluation ^c^
Psychosocial support						
1	5	4	0.80	0.156	0.76	Excellent
2	6	5	0.83	0.094	0.81	Excellent
3	6	6	1.00	0.016	1.00	Excellent
4	6	5	0.83	0.094	0.81	Excellent
5	6	4	0.67	0.234	0.57	Fair
6	6	6	1.00	0.016	1.00	Excellent
Symptom management						
7	6	6	1.00	0.016	1.00	Excellent
8	6	6	1.00	0.016	1.00	Excellent
9	6	6	1.00	0.016	1.00	Excellent
10	6	6	1.00	0.016	1.00	Excellent
11	6	6	1.00	0.016	1.00	Excellent
12	5	5	1.00	0.031	1.00	Excellent
		Average I-CVI	0.93			

I-CVI = item-level content validity index; average item-level content validity index = average I-CVI; ^a^ Pc = probability of chance occurrence [N!/A!(N-A)!]*.5N. N = number of experts, A = number agreeing on good relevance. ^b^ k*=kappa designating agreement on relevance. (I-CVI-pc)/(1-pc). ^c^ Evaluation, kappa values: fair = 0.40–0.59, good = 0.60–0.74, excellent > 0.74.

## Data Availability

The data presented in this study are available on request from the corresponding author.
